# Editorial: Energy metabolism within the skeleton

**DOI:** 10.3389/fphys.2023.1205435

**Published:** 2023-05-22

**Authors:** Bram C. J. van der Eerden, Ryan C. Riddle, Steve Stegen, Michaela Tencerova

**Affiliations:** ^1^ Department of Internal Medicine, Erasmus MC, Erasmus University Medical Center, Rotterdam, Netherlands; ^2^ Department of Orthopaedics, University of Maryland School of Medicine, Baltimore, MD, United States; ^3^ Research and Development Service, Baltimore VA Medical Center, Baltimore, MD, United States; ^4^ Department of Chronic Diseases and Metabolism, Clinical and Experimental Endocrinology, KU Leuven, Leuven, Belgium; ^5^ Molecular Physiology of Bone, Institute of Physiology of the Czech Academy of Sciences, Prague, Czechia

**Keywords:** energy metabolism, bone cells, glucose, fatty acids, amino acids

Following a fracture, an orchestrated sequence of events is initiated *within bone* involving activation of the blood clotting and immune systems, blood vessel formation, cartilage and bone formation and finally bone remodelling to return to the pre-fracture state. This is a prime example when substantial energy metabolism is required at the level of the various cell types present in bone and in the bone marrow. This review Research Topic comprises a set of 6 manuscripts that all recognize in their own way the importance of energy metabolism *within* the skeleton.

Metabolic bone diseases including obesity, diabetes, and osteoporosis are associated with increased risk of bone fractures caused by impaired bone remodeling and function of bone marrow stromal cells (BMSCs) with different underlying mechanisms. These different diseases share features including hyperglycemia and hyperlipidemia defining their pathology, but with different consequences on the skeletal system. Utilization of nutrients by BMSCs has been shown to affect differentiation potential, cell metabolism and bone marrow microenvironment.

Arponen et al. investigated the role of different glucose transporters (GLUT1-GLUT4) in osteoblast differentiation and metabolism using primary female rat BMSCs. This study showed that GLUT1 and GLUT4 are downregulated in osteoblast differentiation, while GLUT3 was not changed during differentiation and GLUT2 was not detected in rat osteoblasts. Using siRNA screening and RNA seq analysis, the authors reported that modulation of GLUT4 expression had a major effect on proliferation and osteoblast differentiation, while GLUT3 mainly influenced cell proliferation. The authors suggested that the glucose transporters are differently involved at various stages of osteoblast differentiation and their functional cooperation is important for the physiological role of osteoblasts.

Bone takes up a considerable amount of postprandial glucose and fatty acids, and these nutrients have been recently shown to fuel specific metabolic pathways in osteoblasts that regulate their function. Certain amino acids, such as glutamine or proline, complement the action of glucose and fatty acids during endochondral ossification, but their roles in the maintenance of postnatal bone mass remain less well understood. In this Research Topic, Shen et al. investigated the role of exogenous proline for osteolineage cells in adult mice through specific deletion of the System A transporter SNAT2 (encoded by Slc38a2) and found that Slc38a2-conditional knockout mice exhibit a significant decrease in trabecular, but not cortical, bone mass. As a mechanism, they proposed that SLC38A2 is necessary for proline and alanine uptake, which in turn is required for proliferation and differentiation of skeletal progenitor cells although the exact metabolic fate of proline and alanine is still unknown. Together, this study clearly demonstrates the importance of SLC38A2-mediated proline and alanine uptake for osteoblast function. Intriguingly, SLC38A2 expression was not only observed in osteolineage cells, but also in growth plate and articular chondrocytes. Whether and how proline uptake regulates their function during bone development, or whether proline metabolism is altered during pathologies such as osteoarthritis are interesting questions that warrant further investigation.

The metabolic requirements of osteoclast-driven bone resorption have yet to receive the same level of attention that osteoblastic bone formation has received. In this Research Topic, Kushwaha et al. examined the requirement for mitochondrial fatty acid β-oxidation by osteoclasts by disrupting the expression of the gene encoding carnitine palmitoyltransferase-2 (Cpt2) in lysozyme-2+ myeloid progenitors that give rise to osteoclasts. Fatty acid oxidation increased during *in vitro* osteoclast differentiation while progenitor cells deficient for Cpt2 failed to form large, multi-nucleated, tartrate-resistant acid phosphatase + osteoclasts. Importantly and in line with these data, female mice deficient for Cpt2 in osteoclasts exhibited a high bone mass phenotype due secondary to reduced osteoclast numbers and activity. These data highlight a key role for fatty acid catabolism in osteoclast maturation and function.

Osteocytes are the most numerous bone cell type in the skeleton and involved in mechanotransduction and subsequent communication with other osteoblast and osteoclast to orchestrate bone formation and resorption, respectively. Given the established involvement of PPARγ as a master regulator of energy metabolism in bone, Lecka-Czernik et al. assessed the role of PPARα in osteocytes by comparing osteocyte-specific and global PPARα knockout mice. It was shown that osteocyte-derived PPARα is vital for the bioenergetics and mitochondrial stress control in these cells and it is up to 40% responsible for the global energy metabolism mediated through PPARα. The contribution of osteocytes to a high energy phenotype typically found in young mice reverts to a low energy and obese phenotype during aging, a phenotype that is shared between the local and global knockout mouse. In contrast, the enlarged bone phenotype and effects on BMSCs and hematopoietic stem cell differentiation following global PPARα deficiency is not observed in the osteocyte-specific model, except increased bone marrow adipose tissue. Apparently, PPARα in osteocytes contributes to systemic energy metabolism by controlling marrow adiposity and peripheral fat metabolism.

Aging, obesity, diabetes and anorexia nervosa are all associated with increased adipogenesis in the bone marrow. In an attempt to identify novel compounds that may counteract enhanced marrow adiposity, Zhang et al. used a drug-repurposing approach to prioritize small molecules employing the connectivity map (CMap). Having selected a set of potential anti-adipogenic compounds, subsequent BMSC-derived adipocyte cultures and transcriptomic profile generation revealed the small molecules emetine and kinetin riboside to potently inhibit adipogenesis. This proof-of-concept study demonstrates that CMap can be used to identify repurposable drugs capable of inhibiting the function of marrow adipocytes.

The review by Thapa et al. reported an interesting overview on the endocrine metabolic regulation on the skeletal system in osteoporosis, with a focus on post-menopausal women. This review focused on the prevalence and different stages of osteoporosis including sexual dimorphism, as higher prevalence in women is caused by loss of the protective role of estrogen. Estrogen levels are key in moderating bone remodeling, keeping a balance between the function of osteoblasts and osteoclasts. This view also interestingly points out the possible role of sex hormones in regulating bioenergetics of BMSCs, which might contribute to manifestation of bone pathology and bone loss induced by osteoporosis. This review provides a perspective focused on the role of gonadal hormones regulating bone cell metabolic potential.

Collectively, the studies within this Research Topic illustrate the need to further study energy metabolism *within* the skeleton and discover mechanisms underlying the pathways involved. Ultimately, this should lead to the development of treatment modalities for metabolic bone diseases, such as osteoporosis, obesity and diabetes.

